# Short-Term versus Long-Term Culture of A549 Cells for Evaluating the Effects of Lipopolysaccharide on Oxidative Stress, Surfactant Proteins and Cathelicidin LL-37

**DOI:** 10.3390/ijms21031148

**Published:** 2020-02-09

**Authors:** Zuzana Nova, Henrieta Skovierova, Jan Strnadel, Erika Halasova, Andrea Calkovska

**Affiliations:** 1 Department of Physiology and Biomedical Center Martin, Jessenius Faculty of Medicine in Martin, Comenius University in Bratislava, 03601 Martin, Slovakia; 2 Division of Molecular Medicine, Biomedical Center Martin, Jessenius Faculty of Medicine in Martin, Comenius University in Bratislava, 03601 Martin, Slovakia; 3 Department of Medical Biology and Biomedical Center Martin, Jessenius Faculty of Medicine in Martin, Comenius University in Bratislava, 03601 Martin, Slovakia

**Keywords:** acute respiratory distress syndrome, alveolar epithelial cells, bacterial lipopolysaccharide, *N*-acetylcysteine, cathelicidin LL-37, surfactant proteins

## Abstract

Alveolar epithelial type II (ATII) cells and their proper function are essential for maintaining lung integrity and homeostasis. However, they can be damaged by lipopolysaccharide (LPS) during Gram-negative bacterial infection. Thus, this study evaluated and compared the effects of LPS on short and long-term cultures of A549 cells by determining the cell viability, levels of oxidative stress and antimicrobial peptide cathelicidin LL-37 and changes in the expression of surfactant proteins (SPs). Moreover, we compared A549 cell response to LPS in the presence of different serum concentrations. Additionally, the effect of *N*-acetylcysteine (NAC) on LPS-induced oxidative stress as a possible treatment was determined. Our results indicate that A549 cells are relatively resistant to LPS and able to maintain integrity even at high LPS concentrations. Their response to endotoxin is partially dependent on serum concentration. NAC failed to lower LPS-induced oxidative stress in A549 cells. Finally, LPS modulates SP gene expression in A549 cells in a time dependent manner and differences between short and long-term cultures were present. Our results support the idea that long-term cultivation of A549 cells could promote a more ATII-like phenotype and thus could be a more suitable model for ATII cells, especially for in vitro studies dealing with surfactant production.

## 1. Introduction

The alveolar epithelium has a key role in maintaining lung integrity and homeostasis. It consists of alveolar epithelial type I cells (ATI) and alveolar epithelial type II cells (ATII). ATI cells are large, flat cells which cover approximately 90% of the alveolar surface, and their main roles are gas exchange and maintenance of fluid balance. A major function of small cuboidal ATII cells is the production of pulmonary surfactant, which is fundamental for preventing of alveolar collapse by reducing surface tension at the end of expiration, and plays an important role in the local pulmonary defense mechanisms in which mainly surfactant proteins (SPs) are involved [1,2]. ATII cells are also the main source of endogenous antimicrobial peptides; among them is cathelicidin hCAP18/LL-37 [3,4]. Therefore, these cells are also referred as “defenders of the alveolus.” Another very important role of ATII cells is the repairing of damaged tissue, as they are capable of self-regenerating and trans-differentiating into ATI cells [5]. Thus, loss of this cell population may be a basis for various pulmonary disorders.

One of the toxic substances damaging the alveolar epithelium is lipopolysaccharide (LPS), also termed endotoxin, which enters the organism as a part of the outer bacterial membrane, contributes to local inflammation and leads to systemic toxicity [1,2]. ATII cells express the functional receptors for LPS, such as toll-like receptor (TLR) 2 and TLR 4 [6]. LPS specifically activates TLRs, leading to activation of the nuclear factor-kappa B (NF-κB) signaling pathway, and secretion of pro-inflammatory cytokines and chemokines, such as interleukin (IL)-1β, IL-6, IL-8, tumor necrosis factor α (TNF-α) and type 1 interferons [7,8], whose overproduction is associated with a development of acute respiratory distress syndrome (ARDS) [9,10]. Endotoxin has been shown to reduce ATII cell viability in animal models of LPS-induced acute lung injury (ALI) [11] and in vitro [12,13]. One of the main factors involved in LPS-triggered epithelial cell death that has been suggested is an augmentation of intracellular reactive oxygen species (ROS) [12,14]. Furthermore, LPS is able to modulate surfactant protein levels in ATII cells, and thus may impair the proper function of pulmonary surfactant [8,13,15,16,17]. All these pathological events possibly lead to a severe lung injury. Therefore, it is necessary to study and understand the molecular mechanisms of LPS action and to find effective medicaments to antagonize the harmful effects of endotoxin. One of the suggested treatments could be an administration of *N*-acetylcysteine (NAC), a substance with strong antioxidant potential [18]. Besides the lowering of cell ROS levels [19], NAC has been shown to suppress the production of IL-1β, IL-8 and TNF-α [20,21].

As the primary human ATII cells are known to lose their phenotype and surfactant synthesis capacity during a standard culture iin vitro [22,23], the human pulmonary adenocarcinoma A549 cell line is extensively used as a model of ATII cell line, despite the fact that their suitability is still being discussed [22,24,25]. It has been suggested that long-term cultures of A549 cells could have more a ATII-like phenotype than short-term cultures of A549 cells. Extended culturing of this cell line leads to its reduced proliferation and cellular “differentiation“, as evidenced by mRNA gene expression profiling, which revealed increased numbers of up- and down-regulated genes shared with primary ATII cells. Moreover, high numbers of multilamellar bodies with similar phospholipid contents to those found in primary lung tissue were found in long-term A549 cells. Another feature indicative of cell differentiation to the ATII-like phenotype was the significant expression of components of complement pathways [22]. Hereby, we aimed to evaluate and compare the effect of LPS on A549 cells in short and long-term cultures by determining cell viability, levels of ROS and cathelicidin LL-37 and changes in expression of all SPs. Moreover, the effect of NAC on LPS-induced ROS production in A549 cells was determined, as we hypothesized that the harmful effect of LPS could be overcome by the use of this antioxidant agent.

## 2. Results

### 2.1. The Effect of LPS on Cell Viability

**Figure 1:**
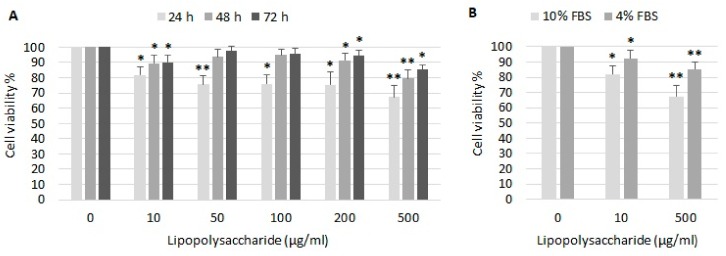
The effect of lipopolysaccharide (LSP) on the relative viability of A549 cells. A549 cells were treated with various concentrations of LPS (**A**) for 24, 48 and 72 h in the presence of 10% FBS, and (**B**) for 24 h in the presence of 10% or 4% FBS, respectively. The relative cell viability was determined by the MTT test as described in Material and Methods. The control value (100%) was determined in cells which were incubated without LPS. Data are presented as means ± SDs from three independent experiments. * *p* < 0.05, ** *p* < 0.01. FBS—fetal bovine serum.

LPS had only moderate effect on viability of A549 cells (Figure 1A). Cell viability decreased to 82% after stimulation with 10 µg/mL LPS, to 75% after stimulation with 50–200 µg/mL LPS and to 67% after stimulation with 500 µg/mL LPS after 24 h. Surprisingly, cell viability was better after longer incubation with LPS. After 48 and 72 h, LPS 10–200 µg/mL had almost no impact on cell viability, and viability decreased to 79% and 85% in cells exposed to 500 µg/mL LPS, respectively. Cell viability after 24 h treatment with LPS in the presence of 10% or 4% fetal bovine serum (FBS) was compared (Figure 1B). Cells cultured in a medium with reduced serum exhibited a lower response to LPS. In presence of 4% FBS, cell viability decreased to 92% and 85% after incubation with 10 and 500 µg/mL LPS, respectively.

### 2.2. Generation of ROS by Epithelial Cells after LPS Exposure

**Figure 2:**
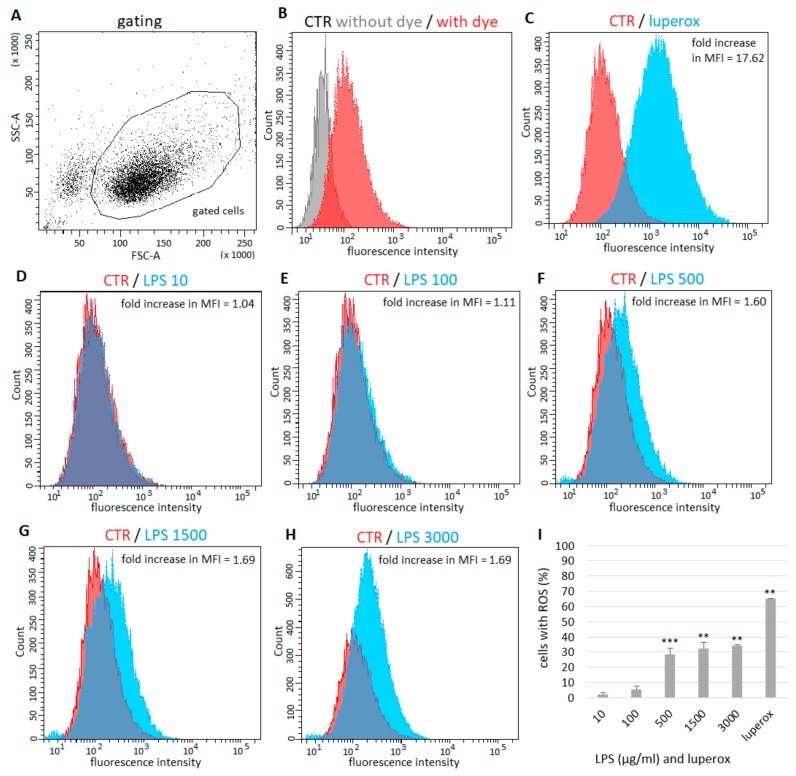
Effect of LPS on generation of ROS in A549 cells. Dead cells and debris were eliminated from analysis by live-cell gating. Along the X-axis is the FSC (Forware SCatter) parameter. Along the Y-axis is the SSC(Side SCatter) parameter (**A**). Unstained and untreated cells were included for elimination of non-specific autofluorescence signal (**B**). Compared to control (H^2^DCFDA-loaded but untreated cells), more than 65% of ROS+ cells were detected in cells treated with Luperox (**C**), no change in ROS levels in cells treated with low concentration of LPS (10 µg/mL) was detected (**D**), 6% more ROS+ were detected after treatment with LPS at 100 µg/mL (**E**) and 28%, 33% and 34% increase of ROS+ cells was observed in cells exposed to LPS at 500, 1500 and 3000 µg/mL, respectively (**F**–**H**). Percentage of ROS+ cells is shown on a graph, untreated cells represent basal (zero) line (**I**). Data are presented as means ± SDs from three independent experiments. ** *p* < 0.01, *** *p* < 0.001. CTR—control, LPS—lipopolysaccharide, MFI—mean fluorescence intensity, ROS—reactive oxygen species.

**Figure 3:**
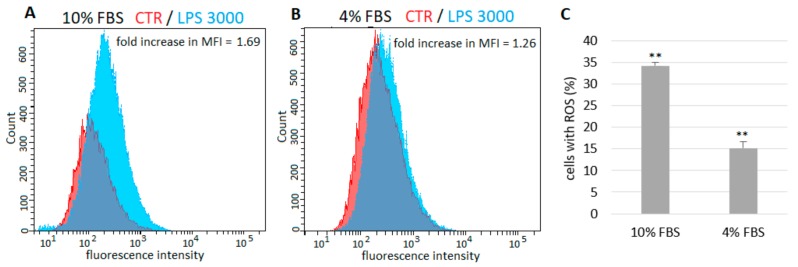
Comparison of ROS generation in A549 cells treated with LPS in the presence of 10% (**A**) and 4% FBS (**B**). Cells cultured in medium with reduced serum exhibited lower response to LPS. (**C**) The percentage of ROS+ fluorescent cells after treatment with LPS 3000 µg/mL. Untreated cells represent basal (zero) line. Data are presented as means ± SDs from three independent experiments. ** *p* < 0.01. FBS—fetal bovine serum, LPS—lipopolysaccharide, MFI—mean fluorescence intensity, ROS—reactive oxygen species

The percentage of ROS possitive (ROS+) fluorescent cells and mean fluorescence intensity (MFI) fold change were determined. Dead cells and debris were eliminated from analysis by live-cell gating (Figure 2A). Unstained and untreated cells were included for elimination of non-specific autofluorescence signal (Figure 2B). In samples containing cells treated with prooxidant agent Luperox, more than 65% of ROS+ cells were detected in comparison with control samples—2′7′-dichlorofluorescin diacetate (H^2^DCFDA) probe-loaded but untreated cells (fold increase in MFI = 17.62) (Figure 2C,I). However, no changes in ROS levels in cells treated with a low concentration of LPS (10 µg/mL) were detected (Figure 2D,I), and only 6% more ROS+ were observed after treatment with LPS at 100 µg/mL (fold increase in MFI = 1.11) (Figure 2E,I). In cells exposed to LPS at 500, 1500 and 3000 µg/mL, the levels of ROS+ cells significantly increased by 28%, 33% and 34 %, respectively (fold increase in MFI = 1.60, 1.69 and 1.69, respectively) (Figure 2F–H). The levels of ROS after treatment with LPS in the presence of 10% or 4% FBS were compared (Figure 3). In samples cultured with a lower concentration of FBS only approximately half the number of ROS+ cells were detected.

**Figure 4:**
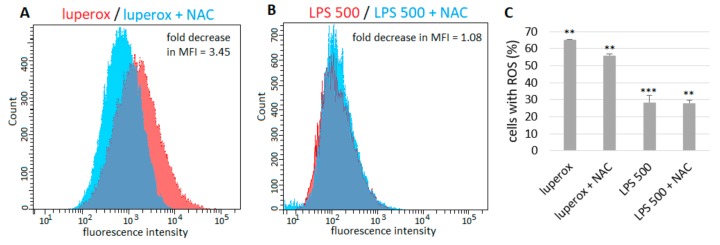
The effect of NAC on ROS+ A549 cells. The numbers of ROS+ fluorescent cells were about 9% lower in samples cultured in the presence of 10 mM NAC compared to cells treated with Luperox only (**A**). NAC did not show any effect on cells exposed to LPS (**B**). (**C**) The percentage of ROS+ fluorescent cells after treatment with luperox or 500 µg/mL LPS alone and in combination with NAC. Untreated cells represent basal (zero) line. Data are presented as means ± SDs from three independent experiments. **p < 0.01, ***p < 0.001. LPS—lipopolysaccharide, NAC—*N*-acetylcysteine.

The number of ROS+ cells was about 9% lower (fold decrease in MFI = 3.45) in samples cultured in the presence of 10 mM NAC compared to cells treated with Luperox only (Figure 4A,C). However, NAC did not show any effect on cells exposed to 500 µg/mL LPS (Figure 4B,C).

Long-term cultivated cells (for 25 days) were observed with high autofluorescence via FITC channel (unstained cells). For this reason, the use of fluorescent dye for the detection of ROS was not possible under these conditions.

### 2.3. Levels of Cathelicidin LL-37

**Figure 5:**
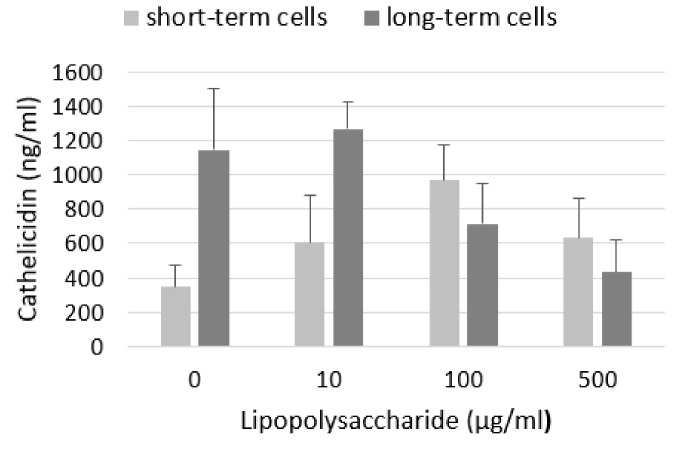
Level of cathelicidin LL-37 in A549 cell lysates. LPS increased level of LL-37 in short-term cells. Long-term cells produce more LL-37 than short-term cells and its level was slightly increased by low concentration of LPS and decreased after treatment with higher concentrations of LPS. Data are presented as means ± SDs from three independent experiments.

We did not detect any LL-37 in the cell culture media of any sample (data not shown). Levels of LL-37 in cell lysates are shown on Figure 5. Compared to control, LPS increased level of LL-37 in short-term cells with the largest increase in cells treated with 100 µg/mL LPS. Untreated cells from long-term culture produced more LL-37 than cells from short-term culture. We observed a slight increase after treatment with LPS at a low concentration (10 μg/mL) but a decrease after exposure to higher concentrations of LPS.

### 2.4. Gene Expression of Surfactant Proteins

**Figure 6:**
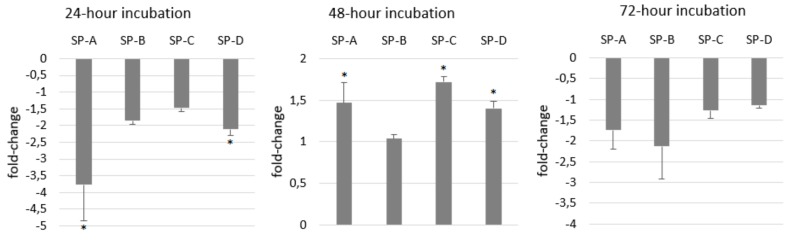
The effect of LPS on surfactant proteins’ (SPs) gene expression in A549 cells. LPS at concentration of 100 µg/mL decreased gene expression of all SPs after 24 and 72 h and increased expression after 48 h. Gene expression in untreated cells represents the basal (zero) line. Data are presented as means ± SDs from three independent experiments. * *p* < 0.05. LPS—lipopolysaccharide, SPs—surfactant proteins.

**Figure 7:**
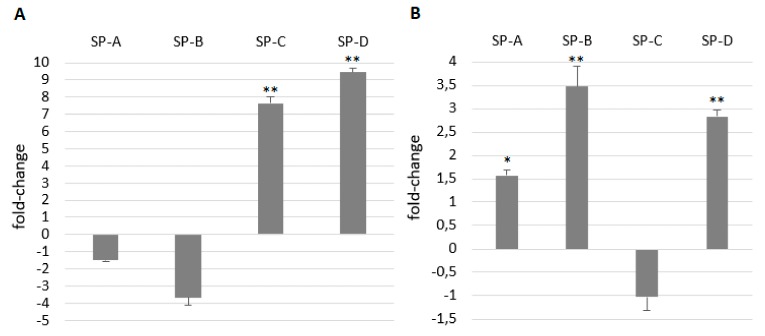
Comparison of SPs’ gene expression in short and long-term cultures of A549 cells (**A**) and the effect of LPS on SP gene expression after 24-h treatment with LPS in long-term A549 cells (**B**). On graph A, SP gene expression in short-term cells represents basal (zero) line. Compared to short-term cultures, gene expression of SP-C and SP-D was much higher in long-term cultures of A549 cells (**A**). On graph B, SP gene expression in untreated long-term cells represents the basal (zero) line. SP gene expression in long-term cultivated cells was enhanced by LPS (**B**). Data are presented as means ± SDs from three independent experiments. * *p* < 0.05, ** *p* < 0.01. LPS—lipopolysaccharide, SPs—surfactant proteins.

ATII cells are typical of surfactant production and secretion of surfactant proteins reflects the metabolism of ATII cells as well as the immunological state of the respiratory system. Therefore, we aimed to monitor the changes in their gene expression after LPS stimulation. At least 1.5-fold decrease was present in the expression of all SPs in A549 cells after 24 h treatment with 100 μg/mL LPS. After 48 h gene expression of SP-A, SP-C and SP-D was slightly increased while gene expression of SP-B did not change (Figure 6). Surprisingly, gene expression of SP-A and SP-B decreased again after 72 h while the expression of SP-C and SP-D almost did not change. Compared to short-term cultures, gene expression of SP-A almost did not differ, SP-B was lower and SP-C and SP-D was much higher in long-term cultures of A549 cells (Figure 7A). SP gene expression of SP-A, SP-B and SP-D in long term was enhanced by LPS and the expression of SP-C did not change (Figure 7B).

## 3. Discussion

ATII cells are considered to be the progenitor population of alveoli and play an important role in innate immune responses of the lungs [2]. Thus, these cells and their proper functions are essential for maintaining lung integrity and homeostasis. However, they may be damaged by LPS during Gram-negative bacterial infection. Therefore, we aimed to assess and compare the effects of LPS on A549 cells (a model for ATII cells) in short and long-term cultures, as Cooper et al. [22] have suggested that long-term cultivated A549 cells are more similar to ATII cells than their short-term cultures.

Literature data about A549 cell responsiveness to LPS is very controversial. Some studies have reported a significant decrease in A549 cell viability by approximately 50% at a very low LPS concentration of 1 µg/mL after 24 h [12,26]. In other studies, a 50% inhibitory concentration of LPS for A549 has been shown for 10 µg/mL [27,28] or even 100 µg/mL LPS [29,30] in 24 h of treatment. Li et al. [31] set as a minimal cytotoxic dose of endotoxin for A549 cells at the concentration of 50 µg/mL. However, cell viability did not fall below 80%. In our study, LPS did not have a great impact on survival of A549 cells. 10 µg/mL LPS only slightly decreased the cell viability after 24 h, which is in line with study of Liu et al. [14] or Huang et al. [29], and even in very high concentration of LPS (500 µg/mL), cell viability decreased only to 67% after 24 h. Surprisingly, better cell viability was observed after 48 and 72 h incubation with LPS compared to 24 h treatment. This may be due to increased cell confluency during measurement, as LPS had no dramatic effect on cell division. According to our personal experience from previous experiments on different cell lines, it is not possible to distinguish the differences in cell viability when cells exceed higher confluence. For this reason, we did not even measure the viability in long-term cultures.

Some research groups used serum-free culture media in experiments with A549 cells and LPS [14,30,32], as it was noted that these cells could respond to endotoxin under serum-free conditions [33]. However, according to Shi et al. [34], the effects of serum-derived factors may remain in the cultures. On the contrary, Thorley et al. [35] did not observe any response of A549 cells to LPS in the absence of serum. Similarly, Tang et al. [36] tested the effect of serum concentration on the viability of A549 cells and they observed LPS-induced cell death only in the presence of serum at a minimum concentration of 5%. One possible explanation could be that A549 cells do not express CD14 protein [35], an important part of LPS signal transduction pathway [2]. Therefore, there is a need for serum as a rich source of soluble CD14 [35,37]. We compared the A549 cell response to LPS in the presence of 4% or 10% serum. Cells cultured in medium with reduced serum exhibited an approximately 50% lower response to LPS, compared to cells cultured with 10% serum. This indicates that the amount of serum in culture medium could influence the interaction of A549 cells with endotoxin.

Another potential reason for the different responsiveness of A549 cells could be the type of LPS used for experiments. We chose LPS from *E. coli*, as it is the most commonly used in studies dealing with inflammation-induced ALI. However, many research groups use different LPS serotypes or LPS from different bacteria and many of them do not specify LPS serotype. It has been shown that LPS substructures could modulate its endotoxic properties, possibly by different interactions of the LPS molecule with the TLR4 receptor complex, leading to different activation of subsequent inflammatory pathways [38,39]. This is supported by studies which have found that structural differences in O-antigen of LPS molecule are able to modulate its recognition and phagocytosis by macrophages [40,41]. According to MacRedmond et al. [42], the bacteria from which the LPS molecule is extracted could also be an important factor. This research group observed better response of A549 cells to *P. aeruginosa* LPS than groups which used LPS from *E. coli*. They have suggested that LPS from *P. aeruginosa* could elicit a stronger response of lung epithelial cells than *E. coli* because *P. aeruginosa* is one of the key respiratory pathogens while *E. coli* is more common in the gastrointestinal and genitourinary tracts. Tang et al. [36] compared viability of A549 cells challenged with LPS derived from *E. coli* or *P. aeruginosa* and observed significant reduction in cell viability after treatment of cells with both types of LPS. However, the authors did not specify the percentage of viable cells after LPS exposure. Thus, it is difficult to recognize which type of LPS elicited a stronger cell death. Moreover, it has been noted that differing cell culture media can have a substantial impact on A549 cell phenotype [22]. Thus, the choice of culture medium may lead to diverse results in studies with A549 cells. Therefore, there is a need to standardize cell culture conditions, including culture medium and serum concentration, cultivation time and type of bacteria in the studies with LPS.

It has been reported that excessive ROS and reactive nitrogen species (RNS) play a key role in pathogenesis of ALI/ARDS [43,44]. The augmentation of intracellular ROS and RNS production has been suggested as one of the main factors involved in LPS-triggered epithelial cell death [12,14]. Considerably increased levels of intracellular ROS and nitric oxide (NO) were also present in A549 cells after LPS treatment [12]. LPS induces ROS production possibly through the activation of NADPH oxidase 2 which is responsible for generating the highly reactive superoxide radical (O_2_^−^) or hydrogen peroxide (H_2_O_2_) as a primary product [45,46]. ROS further enhance the activation of various stress kinases and redox-sensitive transcription factors such as NF-κB [43,47]. Activated NF-κB is further responsible for transcription and activation of inducible nitric oxide synthase [48,49], leading to NO overproduction and synthesis of harmful peroxynitrite (ONOO^−^). In this study we used a H^2^DCFDA probe to detect the level of intracellular ROS. Originally, this probe was thought to be H_2_O_2_-specific, but recent evidence has shown that it is able to detect other ROS, such as hydroxyl radicals (•OH), hydroperoxides and peroxynitrite [50]. We observed that LPS increased ROS levels in A549 cells but only in high concentrations, which is in line with our results from the viability assay. Literature data about ROS generation by A549 cells after LPS treatment differ. In one study, LPS at concentration of 10 μg/mL increased ROS levels only 1.2 times after 24 h [51]; in another one the same LPS concentration for the same time induced a 2.5-fold increase in ROS levels [14]. On the contrary, in the study of Chuang et al. [12], LPS at the very low concentration of 1 μg/mL enhanced intracellular ROS levels by 6.1-fold after 24 h. We also compared ROS levels in A549 cells after LPS challenge in the presence of 4% or 10% serum, and these results corresponded to results from cell viability measurement. In samples cultured with lower FBS, only approximately half of the ROS+ cells were detected. This further supports the idea that culture conditions and amount of serum could modulate the interaction of A549 cells with LPS.

A high level of oxidative stress could be reduced by administration of NAC, a substance with a strong antioxidant potential. Its beneficial effect has been shown in a number of pulmonary diseases, including ALI/ARDS, chronic obstructive pulmonary disease and cystic fibrosis [18,52]. NAC is a thiol, a key precursor of L-cysteine in synthesis of glutathione (GSH) [18,53,54] and the main antioxidant in lung tissue [55] which is necessary for glutathione peroxidase to convert H_2_O_2_ to H_2_O and O_2_ [19,56]. Aside from GSH replenishment, NAC acts as a ROS scavenger [19,53,56] and was shown to suppress NF-κB activation and subsequent cytokine production in cultured cells [57,58] and patients with sepsis [59]. Pretreatment with NAC protected ATII and A549 cells from ROS-induced apoptosis through inhibition of intracellular ROS generation [12,14,60]. The majority of studies are oriented toward the preventive, prophylactic effect of NAC administered before LPS insult. Yet these conditions are not usually present in clinical practice. Therefore, in this study, we simulated the situation where NAC is administered as a treatment after entering LPS into the body. NAC decreased the level of oxidative stress in cells challenged with Luperox. Luperox is an organic peroxide that generates H_2_O_2_ which is effectively converted by glutathione peroxidase in the presence of GSH supplemented by NAC. Thus, on samples with Luperox, we proved that NAC in our experiments is effective. However, NAC did not show any effect on cells treated with LPS. We speculate that ROS generated by LPS could possibly exceed the capacity of superoxide dismutase (SOD), or SOD could be inactivated by persistent inflammation. SOD is an enzyme which is believed to play a main role in preventing oxidative damage in the very first step by catalyzing dismutation of highly reactive O_2_^−^ to O_2_ and less reactive H_2_O_2_ [61,62]. However, decreased SOD activity leads to accumulation of O_2_^−^, which gives the basis for a formation of peroxynitrite or •OH [62]. Peroxynitrite directly reacts with thiols, leading to the formation of disulfide (RSSR), or thiols can be oxidized by radicals formed from peroxynitrite, resulting in the generation of thiyl radicals (RS•) which can react with oxygen and promote oxidative stress by propagating free radical reactions [63,64]. Thus, in this situation, NAC as a thiol could be not-so-effective at lowering oxidative stress. However, more extensive research is needed to prove our hypothesis and to clarify the molecular mechanisms.

As ATII cells are the main source of lung endogenous antimicrobial peptides, including cathelicidin LL-37 [3,4], and it has been shown that the expression of LL-37 can be upregulated during inflammation [65,66], we tested for the presence of LL-37 in short-term and long-term cultured A549 cells and the effect of LPS on its cellular level. LL-37 was present at a low level in short-term cells, and LPS increased its production, which is in line with previous observations [67,68]. Similarly, LL-37 gene expression was upregulated in neutrophils, alveolar macrophages and A549 cells during mycobacterial infection [68,69]. In our study, untreated long-term cultured cells produced more LL-37 than short-term cells, and its level was regulated in different way. We observed a slight increase after treatment with LPS at low concentration (10 μg/mL) but a decrease after exposure to higher concentrations of LPS. It has been shown that LL-37 has a strong binding affinity to LPS, and prevents its binding to the carrier protein (LPS-binding protein) and CD14/TLR4 receptor complex, and thus is able to neutralize LPS’s biological activity [70,71]. Therefore, we speculate that the presence of LL-37 and its increased levels in A549 cells after LPS treatment could partially contribute to already weak response of A549 cells to endotoxin.

It has been shown that LPS can modulate surfactant protein levels in alveolar epithelial cells [2]. In previous studies, SP-A gene expression increased in A549 cells and mice lungs challenged by LPS, while SP-D synthesis remained unaffected by endotoxin [7,16,72]. On the contrary, LPS decreased SP-B expression in animal models of LPS-induced ALI [15,17] and in cultured alveolar epithelial cells [17]. Literature is not fully consistent in case of LPS influence on SP-C expression. One study has reported only a small impact on SP-C expression in LPS injured rat lungs [17]; the other one has found abnormally lower expression of SP-C in LPS-exposed ATII cells [13]. However, the impact of endotoxin on mRNA levels of all SPs in cultured pulmonary epithelial cells has still not been fully investigated. In short-term cultured A549 cells, we observed a decrease in the gene expression of all SPs after 24 h treatment with LPS. That is consistent with our previous experiments, in which at least a 1.5-fold decrease was present in the expression levels of all SPs in the lungs of rats with intratracheal instillation of LPS at the dose 500 µg/kg, while the administration of LPS at the dose 1000 µg/kg even further potentiated this effect [73]. As the molecular mechanism responsible for changes in SP expression, activation of the NF-κB pathway and the subsequent release of cytokines, has been suggested [13,15,72].

Most research groups use short-term cultures of proliferating A549 cells. However, conflicting results about its suitability as ATII cell model have been reported [24,25]. It has been shown that extended cultivation of A549 cells leads to reduced proliferation and promotion of the ATII cell phenotype, as evidenced by high numbers of multilamellar bodies (a typical sign of ATII cells) with similar contents of phospholipids than those found in primary lung tissue [22,74]. Moreover, mRNA gene expression profiling and its comparison with primary cultures of ATII cells suggests a more ATII-like phenotype of A549 cells after long-term cultivation [22]. Thus, we aimed to compare SP gene expression levels and their changes after LPS treatment in short-term and long-term cultured A549 cells. Compared to short-term cultures of A549 cells, gene expression of SP-C and SP-D was much higher in long-term cultures of A549 cells. That could further support the idea that long-term cultivation of A549 cells could promote a more ATII-like phenotype. Thus, long-term A549 cells could be a more suitable model for ATII cells, especially for iin vitro studies dealing with surfactant production. Interestingly, in contrast to SP gene expression in short-term A549 cells, SP mRNA levels in long-term cultured A549 cells were enhanced by LPS. However, the molecular mechanisms need to be investigated.

As the effect of LPS on A549 cells was only moderate, we speculate that alveolar epithelial cells need an interaction with other cells for a better response to endotoxin stimulation. This is supported by other studies, demonstrating that A549 cells cultivated alone were hyporesponsive to LPS at doses up to 100 μg/mL [75,76]. However, in co-culture with human monocytic cells [76] or human peripheral blood mononuclear cells (PBMC) [75], the responsiveness of A549 cells to LPS was markedly upregulated. These results suggest there is a direct interaction between A549 cells and mononuclear cells, which influence a local immune response to endotoxin. LPS-activated mononuclear cells produce cytokines (IL-1β and TNF-α) and chemokines which seem to stimulate pulmonary epithelial cells and amplify its response to LPS, further potentiating inflammatory reactions in the lungs [75,76]. Similarly, an active crosstalk between A549 cells and blood neutrophils was demonstrated. In their co-culture, neutrophils released soluble mediators which acted on A549 cells, but the epithelial cells also influenced the surface expressions of several innate immune receptors on neutrophils in response to endotoxin [8]. Furthermore, the gene expression of SP-B was decreased in cultured human pulmonary epithelial cells after treatment with conditioned medium from LPS stimulated macrophages, whereas in a monoculture of epithelial cells, LPS had not effect [77]. Janga et al. [78] investigated the impact of LPS on monocultures of alveolar epithelial cells and microvascular endothelial cells, and a co-culture of both cell types, possibly imitating a functional alveolar-capillary barrier. Surprisingly, LPS had almost no effect on the barrier properties of epithelial cells in the monocultures. However, barrier properties were reduced and there were significant changes in gene expression in terms of inflammation in the co-culture with endothelial cells or in the epithelial cell monoculture exposed to culture medium from LPS-treated endothelial cells. Thus, the immunological activation of alveolar epithelial cells seems to be at least partly mediated by pro-inflammatory cytokines secreted by LPS-treated endothelial cells. The pathophysiology of ALI/ARDS is complex, and ATII cells seem to be more vulnerable to LPS in the presence of other alveolar capillary membrane-related pulmonary cells than alone.

Taken together, A549 cells seem to be relatively resistant to LPS and are able to maintain integrity even at high LPS concentrations. Their response to LPS is partially dependent on the serum concentration in the culture medium. So, it is necessary to standardize cell culture conditions in studies using LPS. We also modulated the situation when NAC is administered as a possible treatment after LPS enters the body. However, NAC failed to lower LPS-induced oxidative stress in A549 cells in our study. Additionally, we evaluated the effect of endotoxin on SP gene expression and we found that LPS modulates SP gene expression in A549 cells in time dependent manner. Differences in SP gene expression between short and long-term cell cultures were present. Gene expression of SP-C and SP-D was much higher in long-term cultures compared to short-term A549 cells. However, more extensive research is needed to prove the hypothesis that long-term cultured A549 cells could produce a higher amount of pulmonary surfactant than their short-term cultures.

## 4. Materials and Methods

### 4.1. Materials

The A549 human lung adenocarcinoma cell line (CLL-185™), Kaighn’s modification of Ham’s F-12 medium (F-12K medium) and FBS were purchased from American Type Culture Collection (ATCC). Penicillin-Streptomycin Solution was from Biosera. Calcium and magnesium-free Dulbecco’s phosphate buffered saline (DPBS) and TrypLE™ Express Enzyme were from Gibco. LPS from *E. coli* (O55:B5), H^2^DCFDA probe, Luperox^®^ TBH70X (tert-Butyl hydroperoxide solution), sodium dodecyl sulfate (SDS) and RIPA lysis buffer were purchased from Sigma-Aldrich. 3-[4,5-dimethylthiazol-2-yl]-2,5-diphenyltetrazolium bromide (MTT) was from Duchefa Biochemie. NAC (ACC Injekt sol inj) was from Salutas Pharma. High Pure RNA isolation Kit and cOmplete™ Protease Inhibitor Cocktail were from Roche. QuantiTect Reverse Transcription Kit was from Qiagen. TaqMan^®^ Gene Expression Assays for SPs and TaqMan Fast Advanced Master Mix were from ThermoFisher. ELISA Kit for Cathelicidin Antimicrobial Peptide (product number CEC419Hu) was from Cloud-Clone.

### 4.2. Cell Culture and Drug Treatment

A549 cells were cultured in F-12K Medium supplemented with 10% FBS (*v*/*v*), 100 I.U./mL penicillin and 100 μg/mL streptomycin seeded at an optimal cell density of 5 × 10^3^ cells/mL in humidified atmosphere with 5% CO_2_ at 37 °C. The culture medium was renewed every third day. For short-term cultivation, cells were maintained for 3 days at standard cultivation conditions. For long-term cultures, cells were maintained 25 days [22], and culture media were changed every 2–3 days. LPS was dissolved in culture medium and used for the cell treatment in a concentration-dependent manner based on the analysis. In brief, for the cell viability assay—0, 10, 50, 100, 200 and 500 μg/mL; for ROS quantification—0, 100, 500, 1500 and 3000 μg/mL; for analysis of SP expression—0 and 100 μg/mL; and for cathelicidin measurement—0, 10, 100 and 500 μg/mL. For ROS quantification, cells were exposed also to LPS with 10 mM NAC. In the cell viability assay and ROS quantification experiments, cells were treated with LPS in the presence of 10% or 4% FBS, and cell responses to LPS were compared.

### 4.3. Cell Viability Assay

Viability of A549 cells was determined by a colorimetric MTT assay, based on enzymatic reduction of yellow MTT to purple formazan [79]. Cells were seeded in 96-well microtiter plates (1300 cells/well). On the third day, media were replaced by culture media with LPS in varied concentrations in the presence of 10% or 4% FBS, and cells were incubated for 24, 48 or 72 h. After LPS treatment, cells were rinsed by DPBS and further incubated in fresh medium containing 0.5 mg/mL MTT for another 5 h at 37 °C in a humidified atmosphere. Then, SDS (5% (*w*/*v*)) was added to dissolve the formazan product, and after 18 h of incubation, the absorbance was measured at 540 nm (Synergy H1, BioTek). The relative viability of cells was determined as a ratio of optical density of formazan produced by treated cells to optical density of formazan produced by non-treated cells and expressed as a percentage of control. For each incubation time, the optical density value of non-treated control cells was considered 100% of viable cells.

### 4.4. Detection of Intracellular ROS Production

**Figure 8:**
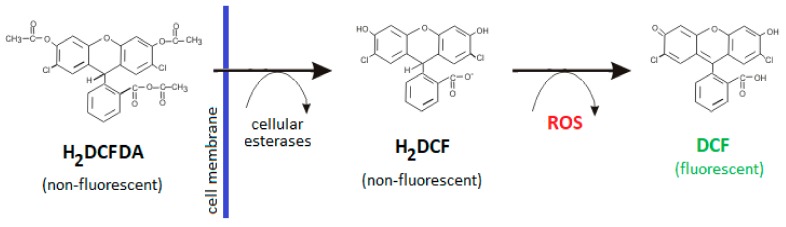
The principle of ROS level measurement using H^2^DCFDA probe. 2′,7′-dichlorofluorescin diacetate (H^2^DCFDA) is a non-fluorescent, cell-permeable reagent which is deacetylated by cellular esterases to a non-fluorescent 2′7′-dichlorofluorescin (H^2^DCF) after diffusion into the cell. H^2^DCF is then oxidizied by ROS into a highly fluorescent 2′,7′-dichlorofluorescein (DCF). ROS—reactive oxygen species.

To determine the level of oxidative stress in A549 cells after LPS exposure, the levels of intracellular ROS were measured using a cell-permeable H^2^DCFDA probe which was intracellulary converted into a highly fluorescent 2′,7′-dichlorofluorescein (DCF) after reaction with ROS [50] (Figure 8). A549 cells were seeded in 6-wells plates (30,000 cells/well) and cultured in media with 10% FBS. On the third day, cells were treated with LPS of varied concentration or LPS with 10 mM NAC in the presence of 10% or 4% FBS for 24 h. Following drug treatment, cells were rinsed by DPBS and incubated in fresh media with reduced serum (2%) containing 5 µM H^2^DCFDA for 30 minutes at 37 °C in humidified atmosphere. Stained cells were harvested, washed and re-suspended in DPBS. Fluorescence intensity was immediately measured via an FITC channel with the FACS Aria II flow cytometer (BD Bioscience). Cells treated with Luperox (500 µM; 1 h) and stained with H^2^DCFDA were used as the positive control. Unstained and untreated cells were included in controls according to the manufacturer’s instructions.

### 4.5. Measurement of Cathelicidin LL-37

Cells were incubated with LPS for 24 h After LPS treatment, both cell culture media and cells were collected. Culture media were centrifuged and supernatants were used for analysis. Cells were washed three times with DPBS, lysed on ice using RIPA with the protease inhibitor cocktail, ultrasonicated and centrifuged to remove cellular debris. The supernatant was used for analysis. Content of LL-37 was measured by ELISA following the manufacturer’s instructions.

### 4.6. Real-Time PCR

A549 cells were exposed to LPS for 24, 48 and 72 h. Total RNA was extracted using High Pure RNA isolation Kit and transcribed into cDNA by QuantiTect Reverse Transcription Kit according to the manufacturer′s instructions. Expression of surfactant protein genes *SFTPA1*, *SFTPB*, *SFTPC* and *SFTPD* was analysed by quantitative real-time PCR (qRT-PCR) using predesigned TaqMan^®^ Gene Expression Assays (*SFTPA1*—Hs00831305_s1, *SF*TPB—Hs01090658_g1, *SFTPC*—Hs00161628_m1, *SFTPD*—Hs01108490_m1) and TaqMan^®^ Fast Advanced Master Mix on ViiA 7 Real-Time PCR System (Applied Biosystems). β-actin was used as a reference gene (Hs99999903_m1). Relative gene expression was calculated by the 2^−ΔΔ*C*t^ method.

### 4.7. Statistical Analysis

Results are expressed as means ± SDs of 3 independent experiments. Statistical analysis was performed using Student′s t-test. A *p*-value of < 0.05 was considered statistically significant.

## Abbreviations

ALI	acute lung injury
ARDS	acute respiratory distress syndrome
ATII cells	alveolar epithelial type II cells
DCF	2′, 7′ –dichlorofluorescein
DPBS	Dulbecco’s phosphate buffered saline
FBS	fetal bovine serum
GSH	glutathione
H^2^DCF	2′7′-dichlorofluorescin
H^2^DCFDA	2′7′-dichlorofluorescin diacetate
IL	interleukin
LPS	lipopolysaccharide
MFI	mean fluorescence intensity
MTT	3-[4,5-dimethylthiazol-2-yl]-2,5-diphenyltetrazolium bromide
NAC	*N*-acetylcysteine
NF-κB	nuclear factor-kappa B
NO	nitric oxide
ROS	reactive oxygen species
SDS	sodium dodecyl sulfate
SOD	superoxide dismutase
SP-A	surfactant protein A
SP-B	surfactant protein B
SP-C	surfactant protein C
SP-D	surfactant protein D
SPs	surfactant proteins
RNS	reactive nitrogen species
TLR	toll-like receptor
TNF-α	tumor necrosis factor α
